# New Method of Administering Quinine

**Published:** 1843-03-18

**Authors:** 


### *New Method of Administering Quinine.*

By Dr. Guastamacchia.

The author's object was to find some method of avoiding the disgust which the bitterness of quinine always excites; and after repeated trials, he says he found it best to dissolve eight grains of the sulphate in half an ounce of rectified spirit, and rub it, in two doses, with an interval of a quarter of an hour between them, along the spine. In intermittent fever this should be done at the beginning of the cold fit; and it very often prevented even a single recurrence of it.—*Brit. and For. Med. Rev. from Il Filiatre Sebezio.* Aug. 1841.

